# Electrochemical communication with the inside of cells using micro-patterned vertical carbon nanofibre electrodes

**DOI:** 10.1038/srep37672

**Published:** 2016-12-01

**Authors:** F. J. Rawson, M. T. Cole, J. M. Hicks, J. W. Aylott, W. I. Milne, C. M. Collins, S. K. Jackson, N. J. Silman, P. M. Mendes

**Affiliations:** 1 School of Pharmacy, University of Nottingham, Nottingham, NG7 2RD, United Kingdom; 2 Electrical Engineering Division, Department of Engineering, University of Cambridge, Cambridge, CB3 0FA, United Kingdom; 3 School of Biomedical & Healthcare Sciences, University of Plymouth, Portland Square, Drake Circus, Plymouth, Devon PL4 8AA, United Kingdom; 4 Public Health England, Porton Down, Salisbury, SP4 OJG, United Kingdom; 5 School of Chemical Engineering, University of Birmingham, Edgbaston, Birmingham, B15 2TT, United Kingdom

## Abstract

With the rapidly increasing demands for ultrasensitive biodetection, the design and applications of new nano-scale materials for development of sensors based on optical and electrochemical transducers have attracted substantial interest. In particular, given the comparable sizes of nanomaterials and biomolecules, there exist plenty of opportunities to develop functional nanoprobes with biomolecules for highly sensitive and selective biosensing, shedding new light on cellular behaviour. Towards this aim, herein we interface cells with patterned nano-arrays of carbon nanofibers forming a nanosensor-cell construct. We show that such a construct is capable of electrochemically communicating with the intracellular environment.

Tools that allow for the study of cell function and identification of the various signalling pathways involved in controlling cellular behaviour are crucial in understanding the manifestation of disease state. Several bio-analytical tools are now available for single-cell analyses, including fluorescent probes, lab-on-a chip technologies, microelectrode-based electrochemical methods and mass spectrometric techniques^1^. Despite important developments, the biochemical processes in a cell can be only poorly quantified, limiting the ability to resolve the dynamic molecular processes that underlie important cell-fate decisions such as differentiation, cell division and cell death^2^. To date, such bioanalytical tools developed are limited in their ability to collect spatial and temporal information^3^,^4^,^5^. For instance, fluorescent probes rely on the formation of a fluorescent product and are therefore not reversible. Thus, they cannot effectively monitor dynamic changes in levels of different intracellular molecules. Detection sensitivity and nanometer-scale spatial resolution are limitations associated with lab-on-a chip technologies, and traditional microelectrode-based electrochemical methods and mass spectrometric techniques. There is a pressing need to develop nanotechnology that can interact with biomolecules within cells on the equivalent molecular scale. Developing technologies with such capabilities will enable researchers to shape our ability to contribute in the understanding of cellular processes that govern a cell’s behaviour.

Some of the common problems associated with fluorescent techniques and the challenges that are yet to be solved in monitoring the dynamic nature of the intracellular environment of cells have been recently highlighted by Spiller and co-workers^2^. It was identified in this work that the development of label-free intracellular detection is of great importance. The development of nanostructured electrochemical sensing systems can address these key issues and offer the capability of measuring electron transfer events occurring from nanomaterials^6^. Since nanomaterials and biomolecules are of similar length scale, this yields the opportunity to interface both entities and electrochemically detect molecular sized species with sub-millisecond time resolution^7^. Thus, nanostructured electrochemical sensing systems are providing the capability to measure events down to an equivalent level to the chemical reactions that occur within cells, enabling a deeper insight into the various cellular signalling pathways^8^.

Electrodes with fabricated nanostructures have been previously interfaced with the inside of cells^4^,^5^,^9^,^10^,^11^,^12^,^13^,^14^,^15^,^16^,^17^. However, given the complexity and diversity of cells, such studies are still very limited in number and so are the opportunities to stimulate and analyse cellular processes, occurring inside cells, with high spatial and temporal resolution. Studies so far have yielded substrates with synthesised vertical silicon nanowires that are able to penetrate mammalian cells without affecting cell viability^9^ and deliver biomolecules such as proteins and DNA plasmids into living cells^10^. Additionally, field effect transducers, based on silicon nanowires and modified with lipid bilayers, could efficiently access the intracellular compartment^11^. Other examples include the intracellular insertion of DNA nanowires, which could electrochemically communicate with a cell^12^. We have recently modified surfaces with chemically self-assembled vertical aligned singled walled carbon nanotubes^4^,^18^ which were capable of communicating with the intracellular environment^4^ and further developed and used to delineate the immune cell function^5^. Others have used a single platinum nanoelectrode and inserted this in to a cell. They subsequently showed that it was possible to measure intracellular reactive oxygen species (ROS)^13^,^14^. Although we are witnessing great progress on the development of intracellular sensing systems over the past few years, the strategies reported to date have their own limitations in particular in terms of spatial and temporal resolution and sensitivity. Taking into account these limitations, it becomes necessary to exploit other nanoscale materials and structures in order to bring new prospects and previously unattainable spatial and temporal resolution and sensitivity to electrochemical detection of intracellular biochemical species.

The ability to grow carbon nanofibres (CNFs) in patterned arrays by plasma enhanced chemical vapour deposition (PE-CVD) with high resolution electron beam lithography to accurately control their length, pattern and position^19^,^20^,^21^,^22^ sets CNFs apart from other nanomaterials, and represents a substantial advantage for high spatial resolution intracellular electrochemical sensing. However, intracellular sensing based on such geometrically tuneable CNFs arrays has not been demonstrated yet.

In the investigation described here, a new generic cell-nanosensor construct (Fig. 1a), based on patterned arrays of PE-CVD synthesised CNFs, for high spatial and temporal resolution and sensitive intracellular detection is introduced. First, our ability to realise different array patterns is demonstrated. The reason for using the different examples of patterns is to provide proof of concept that we can alter patterns readily using electron beam lithography which is important for potential applications. For example, if the intention is to map intracellular signalling events or to reduce/increase current densities to maximize limits of detection then pattern and feature specification becomes important. Subsequently, electrochemical studies were performed to evaluate mass transport behaviour. Lastly, studies focused on determining whether it was possible to harness patterned CNF nanoelectrodes to gain access and electrochemically communicate with the internal environment of macrophage cells.

## Results

### Characterisation of Surfaces

**Figure 1:**
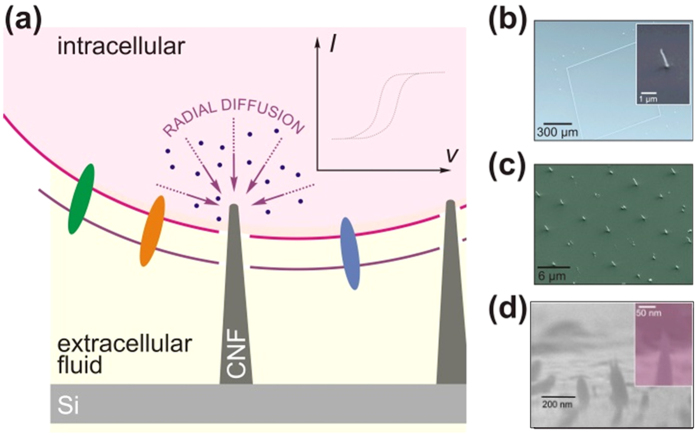
(**a**) Schematic of surfaces and cellular application (**b**–**d**) typical scanning electron micrographs of patterned silicon chips coated with vertically aligned plasma enhanced chemical vapour deposited carbon nanofibres (**a–c**) and for comparison purposes chemically assembled SWCNTs (**d**).

Whilst we have recently reported on a chemical assembled heterogeneous layer of single walled carbon nanotube electrodes (SWCNTs)^4^,^5^, we had no fine control of the coupling process which led to fabrication of a perturbed surface morphology in which we had varying dimensions of coupled SWCNTs, as observed in the SEM images of chemical assembled SWCNTs (Fig. 1d). The fabrication of arrays of forests of SWCNTs using the chemical assembly approach is problematic when interfacing with cells as the number, surface area, and type of SWCNTs that acts as a sensor cannot be controlled with ease. The current output associated with the sensing is critically dependent upon these variables. The overall aim of the current investigation was to establish a nano-patterned electrochemical sensor-cell construct that would permit communication with the inside of a mouse macrophage cell. Our method of tackling this challenge was to use patterned arrays of vertically aligned CNF grown by chemical vapour deposition (CVD). In order to understand the nature of the electrochemical signal in later studies, it was important to electrochemically and physically characterise the surfaces so that we would understand the intracellular response. Therefore, prior to cell dispersion SEM images were taken of CNF patterned arrays and chemically assembled arrays of single walled carbon nanotubes (SWCNTs) (Fig. 1). Different patterns of CNF arrays were fabricated. One of the advantages of using a CVD system, for producing electrode arrays, is the method’s ease of adaptability to tune patterns which will be important for targeting specific areas within cells for intracellular measurements. Consequently, the patterned surfaces consisted of CNFs in a rectangular array with dimensions of 600 μm × 850 μm forming a CNF band thickness of 7.5 μm (Fig. 1b). Within the rectangular pattern we defined a random array of CNF columns that were grown to an approximate height of 500 nm with a diameter of 80 nm (Fig. 1c). Another pattern utilised was an ordered array of CNFs with approximate height of 1 μm with 5 μm spacing between individual CNFs (Fig. 1c) highlighting the ease in adapting different patterned geometries and heights of the CNFs. These were compared to chemically assembled SWCNTs in which diffusion controlled behaviour was observed. This occurs as we see a change from radial diffusion to planar diffusion due to overlapping of diffusion fields, resulting in slower rates of mass transport.

**Figure 2:**
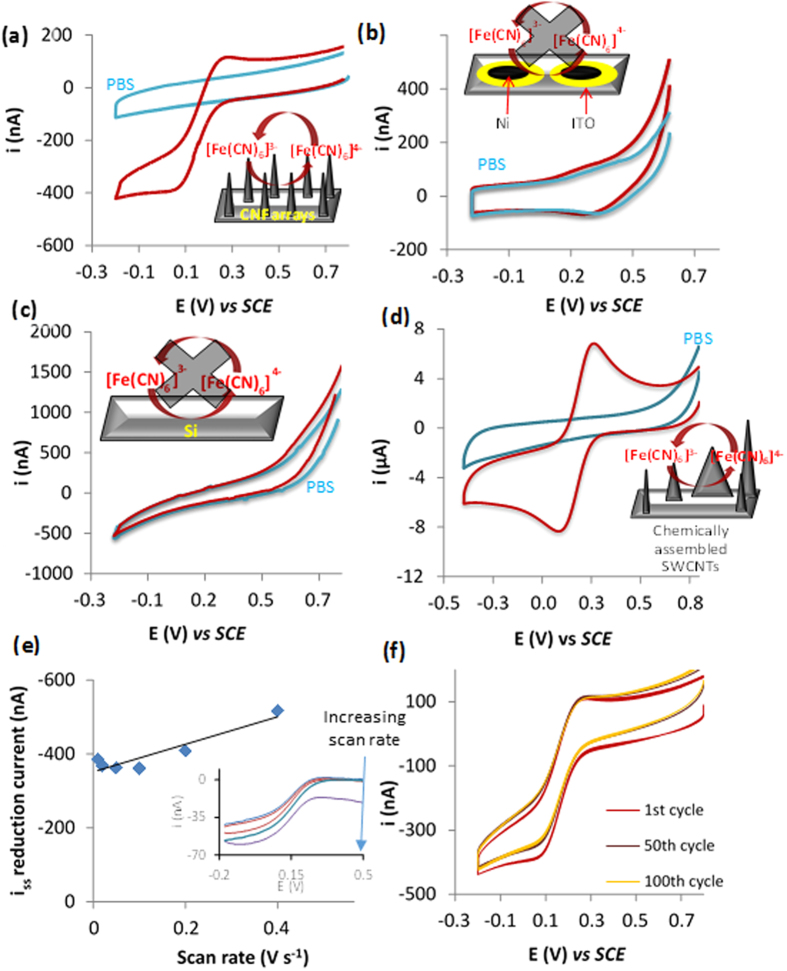
Typical cyclic voltammograms obtained for silicon patterned with random arrays of CNFs (**a**), Si patterned with ITO and nickel (**b**) and bare silicon electrodes (**c**) and chemically assembled SWCNTs (**d**) for solutions of PBS (blue line) and solutions of PBS containing 250 μM ferricyanide (red line) at a scan rate of 0.1 V s^−1^. Voltammograms were performed from a positive to negative direction. Plot of steady state reduction current obtained from voltammograms of 250 μM ferricyanide (Inset) against varying scan rates for CNF patterned electrodes (**e**). Typical cyclic voltammograms obtained at CNF patterned electrodes for solutions of 250 μM ferricyanide at 0.1 mV s^−1^ after 100 consecutive scans (**f**).

Electrochemical characterisation was performed, using cyclic voltammetry employing the well characterised redox probe ferricyanide, at surfaces including those with patterned CNF and those with Ni acting as the control, which was the catalyst used to grow CNFs. Typical cyclic voltammograms obtained for the control solutions of 50 mM PBS and 0.25 mM ferricyanide in PBS obtained at the varying surfaces can be observed in Fig. 2. Cyclic voltammograms recorded with electrodes patterned with random arrays of CNFs can be seen in Fig. 2a and in the presence of ferricyanide a redox couple was observed which results from the 1 electron reduction and subsequent oxidation of the iron centre of ferricyanide. An anodic limiting current was reached at 0.262 V and a cathodic limiting current obtained at approximately 0.027 V *vs* a SCE reference electrode. The observed behaviour can be attributed to ferricyanide as when we compare this to the PBS control no such limiting current was observed (Fig. 2a). This indicates that the PE-CVD CNFs facilitate electron transfer and are well-suited for use as an amperometric sensor. The peak separation potential observed for cyclic voltammograms obtained for solutions of ferricyanide at chemical assembled SWCNTs was 142 mV (n = 4 ±0.04 V) (Fig. 2d) and for CNFs a mean peak separation of 200 mV (±0.025 V n = 4). This means that the electron transfer rates are slightly slower at CNFs when compared to SWCNTs that were chemically coupled (Fig. 2d). Additionally, uncertainties exist in the literature as to the mechanism of the electron transfer when CNF and carbon nanotube modified electrodes are used. There is some suggestion that it is perhaps the catalyst used to fabricate the carbon nanostructures that results in the advantageous electrochemical behaviour^23^. To study this further, equivalent cyclic voltammograms were performed on surfaces with the sputtered, as ITO was the catalyst diffusion barrier, and Ni catalyst (Fig. 2b). No comparable enhancement in faradaic currents was observed when we compare voltammograms obtained for ferricyanide and PBS solutions using silicon modified with ITO and Ni (Fig. 2b) and bare silicon (Fig. 2c). This confirms that it is indeed the CNFs that are facilitating the redox reaction that ferricyanide is undertaking. One central aim of this work was to improve the fabrication procedure and electrochemical behaviour of the electrodes recently fabricated by our group^4^, in which random arrays of chemical assembled vertically aligned SWCNTs were produced. Importantly, we demonstrated that this method resulted in the formation of an heterogeneous surface formed from varying lengths and comparatively misaligned SWCNTs^4^ (Fig. 1d). The improvement would come from controlling the nano-dimensions and alignment of the array by patterning a surface and growing CNFs directly by PE-CVD which also has commercial benefits as this will mean they can be grown en masse. Thus, it was our intention to compare the electrochemical behaviour of ferricyanide when using a random array of CNFs (Fig. 2a) to a chemically coupled arrays of SWCNTs (Fig. 2d). In cyclic voltammograms obtained using the CNF arrays as the working electrode, we observe evidence that mass transport is likely to be a mixture of planar and radial diffusion as there is a pseudo steady-state current obtained. If true microelectrode behaviour was observed it would be expected that we would get a steady-state limiting current and no peaks, as seen by Liu *et al*.^21^.

At the electrode with chemically assembled SWCNTs (Fig. 2d), a peak current is observed in which the current decay after the peak is proportional to t^1/2^, characteristic of planar diffusion kinetics. For the CNF array, this is not the case. The nature of mass transport has changed from planar diffusion, as seen at a macro electrodes or arrays of nanoelectrodes in which the diffusion fields overlap as is the case for the chemically assembled SWCNT array, to one in which radial diffusion is the predominant form of mass transport. The latter is more efficient. An explanation for the pseudo-steady-state currents observed is that with the rectangular pattern we have micro-bands that are larger than 20 μm in one dimension which means there is a contribution of planar diffusion, as previously reported^24^,^25^. However, this behaviour is advantageous as we still note microelectrode behaviour but with larger currents than would be observed at microdisc electrodes which will maximise the sensitivity in later cellular studies. Additionally, the magnitude of the cathodic current obtained for patterned arrays vs chemically assembled surfaces is approximately 400 nA vs 9 μA, respectively. This is due to the larger electroactive surface area that arises from an increase in the number density of SWCNTs on the surface in the chemically-assembled case and thus supports the assertion that at chemically assembled SWCNTs surfaces diffusion fields are overlapping.

To elucidate the exact diffusion behaviour further, cyclic voltammograms were logged in the presence of ferricyanide solutions on CNF patterned surfaces with varying scan rates. Steady-state reduction currents were subsequently measured using the generated voltammograms and these values were plotted against scan rate (Fig. 2e). When cyclic voltammetry is performed at a macroelectrode, where planar diffusion dominates it is well known that the current is proportional to the square root of scan rate. For microelectrode behaviour current is independent of time, and thus scan rate, which occurs due to a change to radial or hemicylindrical diffusion, as observed at the microelectrodes and micro-band electrodes, respectively^24^,^25^. A limiting current based on the latter diffusion characteristics would produce a characteristic horizontal line. On the contrary, Fig. 2e demonstrates that whilst this is the case at very slow scan rates, there is a deviation from this behaviour at high scan rates. However, the limiting current was not proportional to the square root of scan rate, as would be expected for a system under diffusion control, rather, it was linear^26^. Consequently, we conclude that, in the nanostructured CNF case, whilst radial diffusion is the predominant form of mass transport, there is a lesser contribution from the planar diffusion of ferricyanide to the electrode surface which is likely to be from the microband section of the patterned surface.

It was also important to analyse the stability of the electrodes in order to ensure reliable measurements could be acquired once we moved to interfacing the nanostructured electrodes with cells. We therefore recorded 100 consecutive cyclic voltammograms at 0.1 V s^−1^ using ferricyanide solutions. Typical voltammograms obtained during the 100 scans be observed in Fig. 2f. There is little variation (<1%) in the magnitude of the limiting current with scan number. Whilst there is a slight shift in the magnitude of steady-state reduction current, it should be noted that there is also an equivalent shift in the charging current indicating the stability of our surfaces.

### Intracellular Sensing

**Figure 3:**
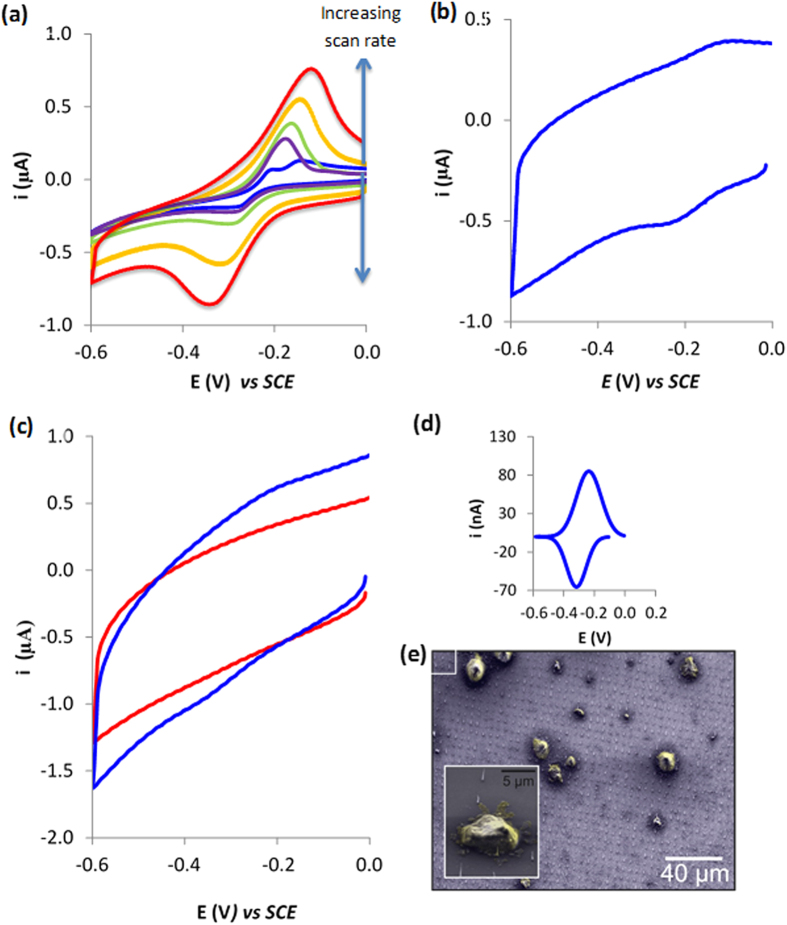
(**a**) Typical cyclic voltammograms obtained with solutions of 500 μM methylene blue for ordered CNF microarray electrodes at varying scan rates including 0.01, 0.02, 0.05, 0.1, 0.2, 0.4 V s^−1^. (**b**) Typical cyclic voltammogram obtained for a ordered CNF microarray with absorbed methylene blue at 1 V s^−1^. (**c**) Typical cyclic voltammograms obtained at electrodes interfaced with cells exposed to methylene blue and either drop coated (red line) or centrifuged (blue line) obtained at 1 V s^−1^. (**d**) Inset is the integrated peak calculated using link fit software) that have been converted into current by multiplying it by the scan rate. (**e**) SEM image of cells centrifuged on to CNF microarray.

It was then the intention to establish whether or not the patterned CNF electrodes could be internalised into the cytoplasm of cells whilst still remaining adhered to the silicon substrate. To this aim, we incubated a well-known biological stain, methylene blue, with cells which to our advantage is also well known to be electrochemically active^4^,^27^. Methylene blue diffuses into cells and is retained. Thus, its redox behaviour acts as an electrochemical fingerprint to identify whether our CNFs could access the cytoplasm. However, prior to running the cells studies it was important to identify the electrochemical behaviour of methylene blue at the CNF electrodes. Cyclic voltammetry was performed at varying scan rates on solutions of 500 μM methylene blue for patterned CNF electrodes. Typical voltammograms obtained can be observed in Fig. 3. At a scan rate of 0.2 V s^−1^, we observe an anodic steady-state current at −0.222 V and a cathodic steady state current at −0.286 V, consistent with a quasi-reversible redox couple (Fig. 3a). There is also a second anodic limiting current at −0.148 V which represents an adsorption peak. At fast scan rates, the peaks become amalgamated as the peak current for an adsorbed species rises quicker than for a diffusion controlled process. To confirm the adsorption, the electrode was removed and rinsed in ethanol and water and placed in fresh PBS and a CV was performed at 1.0 V s^−1^. A signal was observed which arises from adsorbed methylene blue (Fig. 3b). Importantly, this demonstrates that an electrochemical response is observed which can be utilised to establish if CNFs do indeed penetrate the cell interior. Interestingly, for scans above 0.02 V s^−1^, we begin to observe peak currents which arise due to slow electron transfer to the CNFs.

These studies were preceded by investigations to understand the ability of the ordered arrays of nano-patterned CNF electrodes to form a cell-sensor construct that would permit intracellular communication. To this aim, we cultured RAW 264.7 cells and incubated them with 50 μM methylene blue. The cells were rinsed 3 times in PBS to remove solution based methylene blue (MB). We subsequently placed 2 × 10^6^ cells/ml onto the CNF patterned electrodes. After 5 hours, the electrode was removed and cyclic voltammetry performed from 0 V to −0.8 V in order to probe for the electrochemical finger print of the methylene blue. No redox couple associated with methylene blue was observed (Fig. 3c). This was because the CNFs are not traversing the plasma membrane, and thus are not in contact with any methylene blue and as a result no electrochemical signal associated with methylene blue is observed. That is they do not penetrate the plasma membrane without external force or functionalisation. This is supported by our earlier findings for chemically assembled SWCNT, which also needed either to be modified with DNA^4^ or centrifuged with cells^5^ to gain access to the intracellular environment.

We have previously used centrifugal forces to insert chemically assembled CNTs of different lengths inside of the macrophage cells and cell viability was not perturbed^5^. Furthermore, other high-aspect-ratio carbon nanostructures, namely CNFs^28^, have been also inserted into cells in a similar manner without adversely affecting cell viability. In addition, methylene blue reduction has previously been utilized to sense metabolic activity^29^. The fact that the oxidation signal is higher than the reduction (Fig. 3d) indicates that the cells are reducing the methylene blue, and consequently are metabolically active. Thus, this data, in conjunction with our previous data and published examples of CNFs integrated within cells, provides evidence that the structures are not affecting the viability of the cells. Therefore, we used centrifugal forces to insert the CNFs into the cells, which had been previously exposed to methylene blue for 30 minutes. The electrode-cell construct was rinsed 3 times in 50 mM PBS, at 1000 × g. Formation of the nanostructure-cell-sensor construct can be seen from the SEM images of centrifuged cells (Fig. 3e.) Note that the cells were dehydrated in methanol beforehand to fix the sample which explains their unnatural appearance.

Cyclic voltammograms were then performed in PBS on the cell-sensor construct and we observed (Fig. 3c blue and integrated peaks Fig. 3d) a signal that we attribute to the methylene blue redox couple with a reductive peak at −0.33 V and an oxidation peak observed at 0.205 V, and the control in which no external force was applied resulted in no signal associated with MB. The peak potentials observed for methylene blue at SWCNTs modified surfaces was approximately −0.233 V and −0.128 V^2^. These redox peaks were not observed in SWCNT-cell constructs, wherein the cells were not incubated with MB. Thus, these previous studies demonstrate that no intracellular interferences are taking place when considering the assignment of the MB redox peaks. At the CNF surface the peak potentials associated with the redox couple of methylene blue is −0.35 and −0.22 V. The difference can be attributed to using a faster scan rate and the electron transfer rate being slower at CNFs which results in slower kinetics. The reductive current of 69.1 nA (±7.61 nA n = 3) was observed (Fig. 3c, blue) after blank subtraction. This yielded a coefficient of variation value of 12%. This provides compelling evidence that CNF nano-patterned electrodes can sense the intracellular environment.

## Conclusions

In summary, here we provide experimental evidence that electrode carbon nanofibre arrays display electrochemical behaviour for a solution species in which radial diffusion is the main form of mass transport. This is important when designing sensors for intracellular analysis as this will maximise the signal-to-noise ratios in future sensing studies. We show that using centrifugal forces, the CNFs pierce the cell membrane allowing for the intracellular sensing of the biological dye methylene blue. We envisage that this is a generic platform that can be used to monitor intracellular events allowing new biological phenomena to be observed electrochemically. This occurs due to high mass transport rates that occurs at nanoelectrodes, as a result of radial diffusion and lower Ohmic drop which yield increased signal to noise ratios^30^. Additionally, utilisation of patterned CNF arrays whose geometry and inter-electrode pitch can be accurately controlled as shown in these studies opens up the ability of positioning nanostructures at selected spatial regions within cells. This will allow for new studies to understand subcellular heterogeneity and how cellular function maybe related to the geometrical location of a biochemical event.

## Materials and Methods

### Fabrication and Patterning of Surfaces

CNFs were grown as reported elsewhere in our earlier work^31^. In brief, the patterned CNF arrays were grown on degenerately doped Si substrates diced into 10 mm × 10 mm sized chips. Firstly, 80 nm dot arrays were patterned in PMMA by electron beam lithography. An ITO/Ni bilayer catalyst was then deposited by magnetron sputtering. Samples were placed in acetone to lift off residual organics, leaving the bilayer dot stacks. Subsequently, CNFs were grown by loading the sample into a commercial plasma enhanced chemical vapour deposition reactor (Aixtron Ltd.), which was heated to 700 °C (5 °C/s) with the samples exposed to 200 sccm NH_3_ and 50 sccm C_2_H_2_ to stimulate CNF growth. The samples were left to cool under continued flow of ultra-high purity N_2_ for 15 minutes. Two patterns were used and the height of the random array was 1 μm and the height of the ordered array was 500 nm. The methodology for fabricating the chemically assembled SWCNT surfaces has been previously published by our group^4^. Briefly, an ITO conducting substrate was modified with an aryl amine tether layer using well characterised diazonium chemistry. The aryl amine was then used as an anchor for tethering of carboxylated SWCNTs by carbodiimide chemistry.

### Electrochemistry

All electrochemical studies were carried out with a Gamry 600 potentiostat, data acquisition software (Gamry electrochemistry software version 5.61a) and a three-electrode cell consisting of a saturated calomel reference electrode, Pt counter electrode, and a working electrode of either bare lower grade undoped silicon (control), a silicon chip with sputtered ITO and Ni (control) or surfaces with CNFs. The electrochemical area was defined using an O-ring with a diameter of 4 mm.

Cyclic voltammetry was performed to confirm the stability and electroactivity of the surfaces. Cyclic voltammograms were acquired in 250 μM solution of ferricyanide in 50 mM PBS (containing 0.1 M KCl) from a starting potential of 0.8 V and a switching potential of −0.2 V and an end potential of 0.8 V. Cyclic voltammetric studies were also performed to characterize the electrochemical behaviour of methylene blue at the various surfaces. The operating conditions for the cyclic voltammetry was a start potential of 0 V with a switching potential of −0.8 V at varying scan rates from 0.05–1 V s^−1^. Controls of PBS were performed at each surface and 500 μM solution of methylene blue was utilized. For adsorption studies, electrodes were exposed to 500 μM methylene blue for 30 minutes. Prior to performing any scans on the electrodes exposed to methylene blue, surfaces were rinsed in ethanol and water. Peak currents were obtained by subtracting the background using Linkfit software and measuring the cathodic peak current of three replicates.

### Cell Studies

2.5 × 10^6^ Raw 264.7 cells were seeded in three 75 cm^2^ flask containing 18 ml Dulbecco’s modified Eagle’s medium (DMEM), modified with 10% foetal bovine serum (FBS), 1% of penicillin/streptomycin, 2.4% glutamate and 2.4% 2-[4-(2-hydroxyethyl)piperazin-1-yl]ethanesulfonic acid (HEPES). These cells were grown for 2 days at 37 °C in a 5% CO_2_ atmosphere, reaching an approximate 80% confluence at which stage they were harvested. The initial modified DMEM was aspirated off and 3 mL of fresh supplemented DMEM was placed in each flask and cells were detached using a cell scraper. The cell suspensions were pooled giving a total volume of 9 mL. Electrodes modified with CNFs were placed in 50 mL centrifugation tubes and 2 mL of cell suspension at 1 × 10^6^ cell/ml was place in each tube. These were then centrifuged at 1000 × g forcing the electrodes modified with CNFs through the plasma membrane.

## Additional Information

**How to cite this article**: Rawson, F. J. *et al*. Electrochemical communication with the inside of cells using micro-patterned vertical carbon nanofibre electrodes. *Sci. Rep.* **6**, 37672; doi: 10.1038/srep37672 (2016).

**Publisher's note:** Springer Nature remains neutral with regard to jurisdictional claims in published maps and institutional affiliations.
